# Understanding Mammalian Hair Follicle Ecosystems by Single-Cell RNA Sequencing

**DOI:** 10.3390/ani12182409

**Published:** 2022-09-14

**Authors:** Qingbo Zheng, Xiaolan Zhang, Pengjia Bao, Xuelan Zhou, Min Chu, Xian Guo, Chunnian Liang, Heping Pan, Ping Yan

**Affiliations:** 1 Key Laboratory of Yak Breeding Engineering of Gansu Province, Lanzhou Institute of Husbandry and Pharmaceutical Sciences, Chinese Academy of Agricultural Sciences, Lanzhou 730050, China; 2 Key Laboratory of Animal Genetics and Breeding on Tibetan Plateau, Ministry of Agriculture and Rural Affairs, Lanzhou Institute of Husbandry and Pharmaceutical Sciences, Chinese Academy of Agricultural Sciences, Lanzhou 730050, China; 3 Life Science and Engineering College, Northwest Minzu University, Lanzhou 730030, China

**Keywords:** scRNA-seq, hair follicles, single cells

## Abstract

**Simple Summary:**

Single-cell sequencing technology can reflect cell population heterogeneity at the single-cell level, leading to a better understanding of the role of individual cells in the microenvironment. Over the past few years, single-cell sequencing technology has not only made more new discoveries in the study of cellular heterogeneity of other rare cells such as stem cells, but has also become the most powerful research method for embryonic development, organ differentiation, cancer occurrence, and cell mapping. In this review, we outline the use of scRNA-seq in hair follicles. In particular, by focusing on landmark studies and the recent discovery of novel subpopulations of hair follicles, we summarize the phenotypic diversity of hair follicle cells and their links to hair follicle morphogenesis. Enhancing our understanding of the progress of hair follicle research will help to elucidate the regulatory mechanisms that determine the fate of different types of cells in the hair follicle, thereby guiding hair loss treatment and hair-producing economic animal breeding research.

**Abstract:**

Single-cell sequencing technology can fully reflect the heterogeneity of cell populations at the single cell level, making it possible for us to re-recognize various tissues and organs. At present, the sequencing study of hair follicles is transiting from the traditional ordinary transcriptome level to the single cell level, which will provide diverse insights into the function of hair follicle cells. This review focuses on research advances in the hair follicle microenvironment obtained from scRNA-seq studies of major cell types in hair follicle development, with a special emphasis on the discovery of new subpopulations of hair follicles by single-cell techniques. We also discuss the problems and current solutions in scRNA-seq observation and look forward to its prospects.

## 1. Introduction

As a micro-organ embedded in animal skin tissue, the hair follicle plays a key role in the physiological processes of sensing external contact, maintaining body temperature, protecting internal tissues, and preventing foreign body invasion. As a derivative of skin, hair follicles directly affect hair growth, shedding, and regeneration. The hair follicle consists of epidermis and dermis. The epidermis of the mature hair follicle finally differentiates into three enclosed epithelial cylinders, the central most cylinder forms the shaft, the outermost cylinder forms the outer root sheath (ORS) that separates the whole structure from the dermis, and the middle cylinder forms an inner root sheath (IRS) to guide the shaft outward [1]. The hair follicle roots are located in the dermal layer of the skin, which provides important nutrients for the hair, and distributes numerous nerves, muscles, and micro-vessels. The bottom of the hair follicle is the nipple, where the hair grows.

The morphogenesis of hair follicles involves the specialization of various types of cells, among which the specialization of different cell types directly affects the structural composition of hair follicles. The hair follicle consists of hair follicle stem cells (HFSCs), dermal papilla (DP), and IRS, ORS, matrix, hair germ (HG), and other cells. These different types of cell interactions form complex intercellular communication networks, which guide the formation of hair follicle morphology and structure. Therefore, in order to further understand the morphogenesis of hair follicles, it is necessary to enhance the detailed elucidation of this molecular mechanism at the cellular level. Although the hair follicle process is very complex and composed of different types of cells, single cell sequencing technology that can analyze cell heterogeneity has become a popular means of hair follicle research.

Due to the limitations of previous technologies, transcriptome sequencing analysis usually homogenizes the entire organs or tissues of animals, and the sequencing results are the average of all cells, ignoring the heterogeneity of gene expression between single cells, which is challenging for the detailed analysis of the characteristics of rare cells and single cells. The progress of single cell sequencing technology can provide new opportunities for exploring these hidden features [2,3]. At present, single cell RNA sequencing (scRNA-seq) technology has been widely used in stem cell development and differentiation, organ development, tumor field, and disease subtype exploration [4]. However, the number of sequencing cells varies greatly in different studies. The effect of cell number on the construction of the organ single cell transcriptome map is not clear. Hair follicles include a variety of complex cell types, and different types of hair follicle cells have asynchronous development. In the study of hair follicles, single cell sequencing revealed an unprecedented new situation. The single cell map constructed based on single cell sequencing technology can clearly describe the complex cell types of hair follicles, and then study the gene regulation process of specific cell development at the cell level. In this paper, we discussed the main cell types of hair follicle development and the application of single cell transcriptome sequencing in hair follicles in recent years.

**Figure 1:**
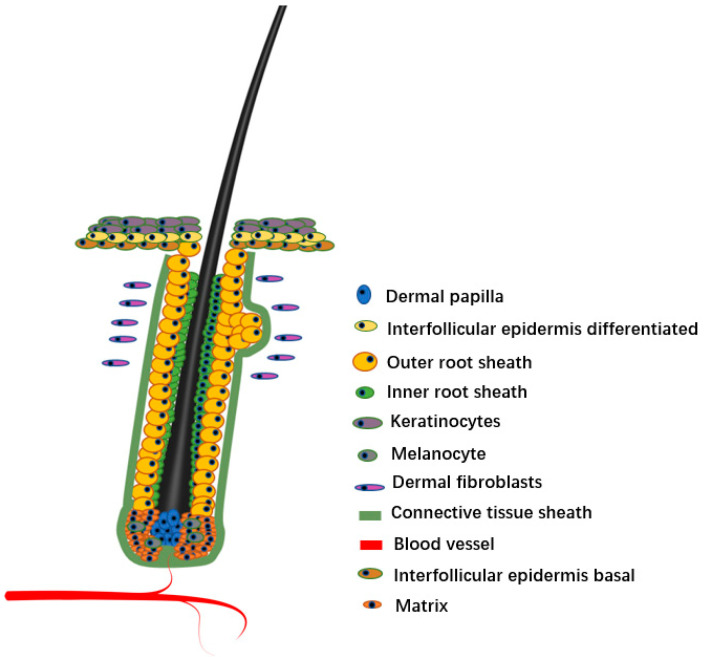
Hair follicle structure.

Hair follicles are composed of a variety of cell lines (Figure 1). Hair follicles can be divided into two types from the origin and function of cells, namely, epithelial cells and dermal cells [5,6]. The epithelial cells are the main body of hair follicles, and they are also the most active part of cell activity in the process of hair formation. The epithelial forms a cylinder with at least eight different concentric layers, including epidermal stem cell, ORS, matrix, and various cell lines differentiated from matrix, including IRS, medulla, cortex, and hair cuticle [7,8]. Although dermal cells do not directly form hair, they are generally considered the ‘signal center’ of hair follicles [9,10]. Generally, dermal cells include two types of cells, DP and dermal sheath (DS). There are differences in the spatial position of DP and DS in the hair follicle, but they seem to be able to convert each other at various stages of hair follicle development, and they both induce hair follicle regeneration and hair regrowth [11]. In this review, we focus on the diversity of hair follicle cells and discuss the progress of scRNA-seq in hair follicle research. We also briefly discuss the problems in the current research on scRNA-seq and the possible future development directions in this field.

## 2. Analysis of Different Cells

### 2.1. Matrix Cell

Hair matrix cells are located in the hair bulb at the lower end of the hair follicle. They are the receiver of the signal of the papilla cells and the precursor cells for the formation of various hair cells. They can differentiate into several different cell types. When the dermal matrix cells were signaled by the DP cells, they began to proliferate and differentiate, and finally differentiated into the IRS and hair shaft. The proliferation and differentiation of hair matrix cells directly affect the integrity and growth of hair structure. Panteleyev et al. [12] proposed the hypothesis of hair follicle predetermination, which indicated that hair matrix cells were differentiated from secondary germs at the end of the telogen, and in the middle of anagen, upon completion of downward growth of the hair follicles, the cells from the bulge region migrated downward along the ORS, survived in the process of programmed apoptosis after catagen, and transformed into HG under the direct influence of follicular papilla, so as to obtain the ability to respond to follicular papilla signals and produce new hair. Hair matrix cells are also the last stop in the formation of hair shafts and IRS. In addition, during the differentiation of hair matrix cells into various cells in the hair follicle structure, hair matrix cells still need to interact with melanocytes and dermal cells, so the normal differentiation of hair matrix cells is the key to the formation of normal hair follicle structure. However, due to the special ecological environment of hair matrix cells, the understanding of the factors affecting the proliferation and differentiation of hair matrix cells is still limited [13].

### 2.2. DP Cell

**Figure 2:**
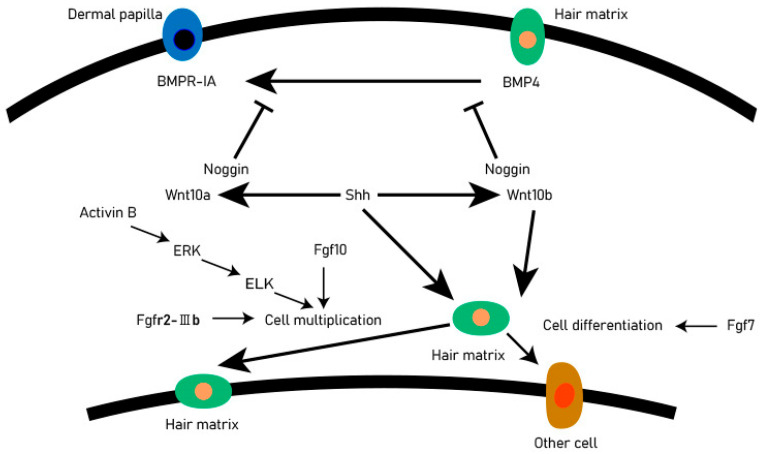
Signal exchange between dermal matrix cells and DP. For each directional solid line, the beginning of the line is the upstream gene, the end is the downstream gene. An arrow indicates that the upstream gene activates the downstream gene, and a blocked line indicates a suppression gene.

The DP provides the guiding signals needed to activate epithelial progenitor cells and initiate hair follicle regeneration. The DP cells play an important role in all stages of hair follicle development and life and are regarded as the ‘command center’ of hair follicles. They determine the thickness, length, and morphogenesis of hair follicles by secreting signaling molecules, such as growth factors and cytokines, and may even determine the periodic cycle of the hair follicle. There are complex and orderly signaling molecules exchanges between hair matrix cells and DP cells, including Wnt, BMP, Shh, and other signaling pathways. These signaling pathways accurately regulate the development of hair follicles through mutual exchange and dialogue (Figure 2). Shh is essential for proliferation of epithelial cells and downgrowths of the regenerating follicle into the dermis [14]. When the Shh gene of mice was knocked out, the mice could normally form placode and dermal condensate, but the hair follicles could not continue to develop backward, resulting in developmental stagnation [15,16]. The activation of the Shh signaling pathway in the early stages of anagen resulted in the expression of Wnt10a and Wnt10b in DP and matrix cells, respectively. The activated Wnt pathway will promote the expression of STAT3, so that the matrix cells can proliferate rapidly. In the early stage of hair follicle formation, the BMP signaling pathway is a kind of inhibitory regulation to promote hair follicle morphogenesis, and it can also prevent the activation of the Wnt signaling pathway and maintain HFSCs in a resting state [17].

At the beginning of the anagen, DP activates the stem cells in the secondary hair germ, causing follicles to regrow downward, however, at the catagen, the epithelial cells at the base of the hair follicle undergo apoptosis, while DP remains intact and is pulled or migrates upwards until it stays next to the stem cells in the hair follicle bulge [18]. The DP experienced periodic changes in volume and histological appearance. In most stages of hair cycle, the DP seems to be in a relatively dormant state. However, in the phase of anagen Ⅳ, DP cells proliferated. The DP is very important for determining the number of basal keratinocytes on the basement membrane and the diameter of hair products. And DP regulates the activity of basal epidermal cells near and on the basement membrane through a series of signaling pathways and interaction between signaling molecules. In DP cells, the notch signaling pathway activates Wnt5a expression by binding to the RBP-Jk promoter region. Studies have found that Wnt5a is an essential downstream mediator of Notch-CSL signaling, impinging on expression in the keratinocyte compartment of Foxn1, a gene with a key hair follicle regulatory function, and Wnt5a can regulate the Notch signaling pathway by regulating Foxn1 gene expression, and Foxn1 plays an important role in the differentiation of hair follicle keratinocytes and the pigmentation of melanocytes [19].

Botchkarev et al. [20] discovered that BMP4 plays the role of inhibiting molecules in telogen after receiving the BMP4 antagonist Noggin that will promote the conversion process of the hair follicles from telogen to anagen, in the early stages of growth. The BMPR-IA in the dermal papilla is located in cells near the club hair. The activation of the Shh signaling pathway leads to the expression of Wnt10 a and Wnt10 b in DP and hair matrix cells, respectively, which makes hair matrix cells proliferate rapidly, and the activin B can regulate the proliferation of hair matrix cells through ERK signaling [21]. The DP cells regulate hair follicle development and secondary hair growth [18], and the quality of villi produced in animals is significantly relational to the secondary hair follicles [22]. Through scRNA-seq, DP cell lineage differentiation trajectories can be constructed to reveal key genes and functions involved in cell fate determination [23]. It is expected that in the future, scRNA-seq can comprehensively analyze the fate regulation process of major cell types during animal hair follicle development, provide reliable candidate genes for animal breeding work, improve the yield and quality of animal villi, and find some potential solutions for hair loss.

### 2.3. Melanocyte

Mammalian hair coat color mainly comes from pigments synthesized by mature melanocytes in the hair matrix of the hair bulb. Melanocytes are mainly concentrated in hair follicles and the epidermis during embryonic development in vertebrates. Hair follicles and sweat glands are called the repository of melanocyte stem cells (MSCs). During the hair follicle growth cycle, MSCs migrate downward to the dermis, proliferate and differentiate into melanocyte cells (MCs) located above the papilla [24]. Melanocytes are positioned on the basal layer, these produce melanic pigments which are transferred to adjacent keratinocytes in melanosome organelles, and which give color to the epidermal tissues produced [12]. Melanocytes in the hair matrix proliferate and differentiate during anagen and transfer melanin pigment to hairs, then stop pigment production during the period of hair follicle catagen, and die due to apoptosis [25]. Therefore, when the hair follicle is in anagen, the melanin deposition is relatively high, and the melanin content gradually decreases with the shedding of the hair shaft. The epithelial column of catagen hair follicles and the capsule of telogen, possibly along with some inactive melanocytes and other melanocyte progenitors, are involved in the secondary hair germ [26,27]. Melanocytes proliferate and migrate to the hair bulb, producing melanin around DP and transferring to keratinocytes in growth of the growing shaft. Therefore, melanin was dense at the junction of the hair follicle base and DP. Melanin synthesis and transfer are regulated by cutaneous signal transduction pathways, such as dependent and independent on receptors, acting through intracrine mechanisms, and being modified by hormone signals [28]. DP cells can affect the proliferation, differentiation, and migration of melanocytes, and affect pigment formation and hair coat color [29]. In the anagen hair follicle, melanogenic active cells are only located in the hair follicle bulb and directly involved in hair shaft pigmentation [26,30,31]. Kwon et al. [24] showed that melanocytes harvested from plucked hair follicles may potentially serve as a renewable source of pigment-producing cells for the treatment of hypopigmentation. Melanocytes in the hair bulb synthesize melanin granules. The number, character, and distribution of melanin granules determine the color of mammalian hair.

### 2.4. IRS Cell

The IRS is located between the hair stem and the ORS, consisting of Henle’s layer, Huxley’s layer, and the IRS cuticle, which is the cell group that determines the formation of hair fibers [32]. In Henle’s layer, keratins are the first to fully keratinize to support hair shape. Since the slow growth rate of Henle’s layer is the earliest keratinized region, it has the effect of protecting the other two layers of IRS and supporting hair shape [8]. The IRS encloses the hairy stem, starting from the upper hair bulb ending at the opening of the sebaceous gland. During the anagen, the HFSCs located in the bulge of the hair follicle were activated and began to proliferate. Subsequently, the HFSCs migrated downward along the ORS into the hair bulb to form hair matrix cells. After rapid proliferation, they began to differentiate and migrate upward to form six cell lines of IRS and hair shaft, namely, Henley, Huxley, and cuticle cell lines, belonging to IRS, and hair cuticle, cortex, and medulla cell lines, belonging to the hair shaft. With the differentiation of all kinds of cells constituting the hair follicle, hair consisting of tight tissue intermediate filaments formed by physical crosslinking of cystine-rich keratin gradually formed. In the process of hair shaft formation, in the IRS, keratinized cells begin to form various concentric circular cells in different terminal differentiation processes, such as hair medulla cells, hair cortex cells and so on. These cells are compressed orderly and tightly to form hair. With the continuous proliferation and differentiation of cells, hair grows continuously and extends from the body surface. Outside the IRS, the ORS cells differentiated from keratinocytes tightly enclose the entire hair follicle [33,34].

The expression of Dlx3, FOXN-1, and HOXC-13 transcription factor plays an important role in regulating the periodic changes of hair follicles and the control of the hair matrix keratinocyte differentiation toward the hair shaft and IRS [35,36]. Human genes trichohyalin (TCHH), peptidylarginine deiminase 3 (PADI3), and transglutaminase 3 (TGM3) could hinder the hardening process of the IRS, resulting in uncombable hair syndrome (UHS) and one of the reasons for hair drying and frizzing [37]. In addition, the latest research showed that there is a spatial structure called Flügelzellen between the Henle’s layer and Huxley’s layer, particularly mentioned is the so-called Flügelzellen, i.e., Huxley cells, where horizontal cell extensions that pass through the Henle’s layer, adjoining the accompanying layer and forming desmosomal junction with surrounding cells, these structures are predicted to strengthen and stabilize IRS, and have an important impact on the formation of hair curl [8,38].

### 2.5. DS Cell

The DS is a layer of connective tissue sheath between the ORS and the dermal layer, which is the skin cell group derived from the mesenchymal source between the DS and the ORS. Numerous studies have identified DS as one of the main candidates for cell-based therapies to reverse hair loss [39]. Surrounding DS may have a distinct precursor cell group, and there may be functional overlap between DS and DP cell groups, and transplanted DP cells and dermal sheath “cup” cells also have the equal ability to form DP and induce hair follicles [40]. When DS cells and matrix cells are mixed and transplanted into the ear trauma of mice, new hair can be induced [41]. When DS cells in the lower part of the beard are transplanted into the ear trauma or plantar of SCID mice, new hair can also be induced [42]. When human DS cells are transplanted into another individual arm, it is found that DS transplantation does not cause immune rejection, and can induce new hair [43], indicating that DS cells can induce new hair without causing immune rejection after allogeneic transplantation. Rahmani et al. [44] found that DS contains a self-renewing cell population that is kept in the territory of mesenchymal niche in successive hair follicle cycles, and at the beginning of anagen, HFSCs will regenerate a new DS and repopulate the DS and the DP with new cells. When the hair follicle root was removed, DS was involved in the repair of DP [45].

Nicholas et al. have demonstrated that DS is a kind of smooth muscle, which can provide power for the regression movements of hair follicles in catagen, and the force pulls DP through tethered-like epithelial cells and wrapped in DP in the form of a hollow sleeve, which plays an important role in the generation of new hair shafts in the next cycle [46]. The characteristics of smooth muscle alpha-actin specifically expressed by DS are related to its shrinkage function, and the contraction function of DS may be related to the control of hair follicle shortening and hair fiber movement in hair cycle [47]. After the hair follicle root was removed, the epithelial cells were filled into the extracted hair stem cavity and formed irregular protuberances to the proximal end. The DS cells were activated and migrated to the lower end of the residual hair follicle. Subsequently, the epithelial cells of the proximal protuberance moved to the distal end, leaving a hanging glass membrane structure. The DS cells further migrated into the glass membrane and entered the extracted hair stem cavity. The new DP was formed and gradually expanded, and the hair fibers began to form [45]. Therefore, cell-based therapy using DS cells to enhance hair regeneration potential is an appealing possibility.

## 3. Single Cell RNA Sequencing Technology

The scRNA-seq technology is a sequencing method that can perform single-cell sequencing of nearly 10,000 cells and can identify the transcription characteristics of various cell types in biological tissues and comprehensively reveal the heterogeneity of gene expression between cells [48]. James et al. [49] and Iscove et al. [50] took the lead in sequencing an entire transcriptome at the level of a single cell. They used in vitro transcription linear amplification and PCR exponential amplification to expand the complementary DNAs (cDNAs) of an individual cell. The earliest report of scRNA-seq was, in 2009, the ‘Nature Methods’ reported single cell transcription map of mouse blastomere stage [51]. Subsequently, more and more scRNA-seq sequencing schemes were widely used in basic scientific research, which played an important role in discovering new heterogeneous cell types and tracking the dynamic development trajectory of cells.

## 4. New Technique for Anatomy of Hair Follicle Development at Single Cell Level

Development is driven and controlled by temporal and spatial changes in gene transcription, followed by translation of the resulting messenger RNAs (mRNA) into proteins [48]. Although substantial progress has been made in hair follicle biology in the past few years, many studies are descriptive, mainly because the definition of various cell types during hair follicle formation is not accurate enough, and there are different types of hair follicle asynchronous development during hair follicle morphogenesis [52]. In the analysis of single-cell sequencing data, accurate cell classification is an important basis for data analysis. However, manual annotation of cell marker genes of different cell types is still a rather difficult and very important task in the data analysis process. Only the precise identification of cell types can clarify the relationship between cells, especially when the differentiation trajectory is constructed. Currently, several websites and software have been developed to assist in the identification of cell types, such as CellMarker (http://xteam.xbio.top/CellMarker/) (23 August 2022) and SingleR (https://github.com/dviraran/SingleR) (23 August 2022), etc. Table 1 also shows some examples of hair follicle marker genes. However, these results can only be used as a reference, and some of these marker genes are used in more than just one hair follicle cell annotation, for example, the marker gene KRT10 is strongly enriched in the interfollicular epidermis, the upper hair follicle, and keratinocytes. Interestingly, these cells belong to the epithelial cell line, indicating that there are many similarities between different cells in the epithelial cell line. Therefore, it is particularly important to use single cell sequencing technology to draw the single cell transcription map of the induction stage, organ formation stage, and cell differentiation stage, during hair follicle development. At the same time, in the process of using marker genes to annotate cells, it is still necessary to use as many markers as possible to identify cell categories.

In recent years, the number of articles using scRNA-seq to analyze the process of hair follicle differentiation has also increased. Khusali et al. used scRNA-seq to distinguish different transcription states in embryonic skin, deduced the transcription state sequence passed by dermal condensates (DC) cells, and found the inference path of molecular state leading to DC cell differentiation, and it revealed that the maturation of DC-related transcription required the conduction of Wnt/β-catenin signal, and clarified that DC cells were descendants of DC progenitor cells highly propagated at telogen [77]. Mok et al. [81] used scRNA-seq to establish the developmental trajectory of DC lineage from fibroblasts, and found that from fibroblasts to DC, there were four stages: fibroblasts, pre-DC, DC1, and DC2. In murine, use of scRNA-seq to analyze hair follicles by Joost and coworkers was published in ‘Cell System’ in 2016 to analyze the heterogeneity of hair follicles in adult murine, which was the first use of scRNA-seq to study cell heterogeneity at the transcriptional level of telogen epidermis, through the analysis of 1422 single cell transcriptomes, 25 distinct populations of interfollicular and follicular epidermal cells were identified, and their specific gene expression profiles were described [74].

Rie et al. used scRNA-seq to identify the unique cell types from follicular-enriched scalp grafts in human hair follicles, and 23 primary cell clusters were obtained, and association of specific cell subsets with known molecular characteristics of common skin diseases was explored; they confirmed previous murine and human studies and provided new insights into the differentiation and pathogenesis of the epidermis and hair follicles [64]. The scRNA-seq of HFSCs revealed five major HFSC populations and new markers, introduced the molecular heterogeneity of HFSCs in the self-renewal stage, and proposed the potential different functions of ORS and bulge subpopulations [63]. Christian et al. used scRNA-seq to study the diversity of skin wound fibroblasts, and found 12 wound fibroblast clusters, and some clusters may represent a continuous differentiation towards the contraction phenotype, while other clusters seem to represent different fibroblast lineages, some subsets express hematopoietic marker genes, indicating that they are of myeloid origin, using bone marrow transplantation and pedigree tracking based on Cre recombinase, it was confirmed that hematopoietic lineage cells produced myofibroblasts and rare regenerative adipocytes [82].

Chae et al. revealed by scRNA-seq that activation of the Sonic hedgehog pathway regenerates a renewable dermal niche called the dermal papilla, explaining its necessity and adequacy for new hair follicles, and revealing that activation of Shh signaling in Wnt active cells promotes the fate of the dermal papilla in scar wounds [83]. Ahlers et al. [62] performed scRNA-seq of skin tissues from different ages and described the characteristics of human DS at the single cell level and found that DS secretory protein Activin A had paracrine effects on keratinocytes and dermal fibroblasts and promoted proliferation. scRNA-seq was used to decipher the functional heterogeneity of skin fibroblasts. Ge et al. [78] used the scRNA-seq sequencing platform to systematically analyze the early morphogenesis of hair follicles in northern cashmere goat fetuses at induction (embryonic day 60; E60), organogenesis (E90), and cytodifferentiation (E120) stages at the single cell level for the first time. The dermal cells, epidermal cells, keratinocytes, DP cells, hair shaft cells, endothelial cells, pericyte cells, muscle cells, and macrophage cells were successfully identified, and the molecular characteristics of each type of cell were described in detail. A recent scRNA-seq study revealed that DP fibroblasts have larger transcriptome differences in the anagen phase compared with the telogen phase of the hair follicle, significantly upregulating a variety of signaling molecules to promote hair regeneration, of which SCUBE3 is only expressed by DP fibroblasts in anagen, but not in the telogen hair follicles, when microinjection of SCUBE3 protein can activate new hair growth [58], which provides a potential way to solve the problem of hair loss.

## 5. Discovery of New Heterogeneous Cell Types by Single Cell Technology

The scRNA-seq technology plays an important role in discovering new cell types, using transcriptome differences between different cells to discover new cell types that have not been previously discovered. Especially in the case of a small number of cells, scRNA-seq has great advantages in analyzing potential small or rare cell groups. Transcriptome analysis of a single cell greatly promotes the dissection of gene expression networks in rare cell types, and, more importantly, helps to identify new cells in these cell groups [84]. Macosko et al. [85] analyzed the mRNA expression in thousands of single cells by encapsulating cells in tiny droplets, and identified 39 cell groups with different transcriptions, revealing the known retinal cell categories and the gene expression profile of new candidate cell subtypes, meanwhile, it was proved that if the complete transcripts were evaluated, it was possible to reveal the new cell type specificity based on the same type variants. scRNA-seq was performed on human blood to expand one plasmacytoid dendritic cell and two conventional dendritic cell populations of human blood into six dendritic cell populations, and four monocyte subtypes were identified: two known subtypes, two new subtypes that have not yet been functionally characterized, and rare cell type AXLSIGLEC6 cells; the existing classification was improved, and the precursor of cDC in blood was determined [86]. Similarly, based on several specific markers, Ductertre et al. [87] revealed the distinct subsets of type 2 conventional dendritic cells, and identified the FLT3L-response IRF4CD14 type 2 conventional dendritic cell subset, and found that the subset showed a pro-inflammatory function in the blood of patients with SLE. Montoro et al. [88] identified a rare cell type, the Foxi1 pulmonary ionocyte, from mouse tracheal epithelium using scRNA-seq and found that the gene expression of this cell is related to special ion transport regulation.

Before the advent of scRNA-seq technology, the specialization process of DC was only generally divided into a stage; using scRNA-seq technology, a precursor stage of DC cells, namely pre-DC cell stage, was newly discovered [81]. Vorstandlechner et al. [72] used scRNA-seq to analyze the heterogeneity of human skin fibroblasts and identified six fibroblast clusters. They found that each subclass had specific biological functions, and the newly identified fibroblast subclass did not overlap with the markers commonly used to identify papillary and reticular fibroblasts. The application of scRNA-seq in mammals indicates a comprehensive parsing of new and existing cell groups. Other studies have enriched specific cell groups, among which DP and DC have been extensively studied, due to their important biological effects. As methodology matures, scRNA-seq technology will be more widely used in the coming years. Single cell sequencing of the development process of various tissues and organs provides a new technical means for finding new cell types and marker genes.

## 6. Future Prospects of scRNA-seq in Hair Follicle Development

Obviously, with the development of the sequencing technology, scRNA-seq technology has made significant progress in the past decade and has been applied to many fields. With the continuous improvement of the methodology, our understanding of cell interaction in hair follicle development has been enhanced, and impressive progress has been made. scRNA-seq enters a seemingly mature stage, but there are also some limitations. For example, scRNA-seq requires the preparation of single cells in cell suspension and the construction of a single cell library by single cell separation technology. Therefore, in this process, the spatial location information of cells will be lost, and spatial heterogeneity is the key feature of organ function. The location information of cells is very important for the study of the cell fate regulation mechanism and the cell lineage generation process. Therefore, although the scRNA-seq dataset tells cells what happened and the molecular relationship between them, we do not know whether the cells obtained by sequencing are closely linked or far apart in the original sample, or whether organ development is related to specific tissue structure or two specific cell distances [89]. Although the known marker genes were used to identify cell types, the spatial background of tissue genes was obtained by restoring scRNA-seq from the spatial localization of known primitive cells. However, the number of target genes that they can detect is still significantly insufficient compared with the high-density gene information in cells.

In order to simultaneously obtain the transcriptional heterogeneity and spatial location information of cells, spatial transcriptomics (ST) technology emerged. At present, there is a wide range of commercial space transcriptomics technology, such as ST technology of 10×Genomics company. This technology provides high quality transcriptome data and their complete two-dimensional location information by locating frozen tissue sections on special carrier chips, arranged with reverse transcription primers and a unique positioning bar code array. At present, ST technology has been used to study the spatial consistency of gingival tissue [90], heart tissue [91], and melanoma [92]. In 2016, Stähl et al. developed the ST technology, which could visualize and quantitatively analyze the transcriptome in a single tissue slice with spatial resolution, high-quality RNA-sequencing data were displayed by positioning histological sections on the array of reverse transcription primers with a unique location barcode, and the two-dimensional location information of mouse brain and human breast cancer was revealed; the principle was that the thin tissue was placed on the slide containing reverse transcription primers, and the slide size was 6.2 mm × 6.6 mm. The slide was composed of 1007 spots, and each spot contained a large number of oligonucleotide chains and specific marker chains, the diameter of each spot was 100 μm, and a center-to-center distance was 200 μm [93]. In 2019, extensive studies were been carried out, the highest resolution can reach 6.1 × 6.5 mm^2^ capture area, which can contain 1007 spots, the diameter of each site is 100 um, and the spacing is 200 μm [94]. Wu et al. conducted spatial transcriptome sequencing in 2021, with a maximum resolution of 5000 spots per 6.5 × 6.5 mm^2^ capture area, each defined by a fiducial frame + capture area is 8 × 8 mm^2^, with a diameter of 55 μm per spot [95].

Space transcriptomics will become an important supplement to scRNA-seq in the future [89]. Therefore, the application of ST technology in the study of spatial heterogeneity of hair follicles still has great room for improvement and application. It is estimated that about 50% of men and 25% of women worldwide suffer from hair loss at the age of 50 [96]. The goal of hair loss treatment is to prevent hair loss and promote regeneration. Hair transplantation is an effective treatment for hair loss. However, for patients without sufficient autologous hair follicles, transplantation of heterologous hair follicles is still ineffective [97]. Therefore, the ultimate goal of the study on hair follicle development is to understand the potential mechanism in order to treat alopecia by inducing hair production by autologous hair follicle cells. The unique role of different cells in hair follicle development is increasingly understood by scRNA-seq technology. With the development of scRNA-seq technology, we hope to reveal the molecular mechanism of hair follicle development and alopecia-specific genotype and serve the treatment of alopecia. In order to solve this problem, cell type identification, pseudo-time analysis, inter-cellular ligand-receptor interaction, and reconstruction of specific gene regulatory network will be extremely important areas for future scRNA-seq research on hair follicle development. At present, the pathogenesis of alopecia is still unclear. The understanding of hair follicle structure, abnormal hair papilla cells, and HFSCs before hair loss is still severely limited. The recording of molecular functions in different cell groups and the discovery of new cell subsets may reveal the most easily handled opportunity for inducing hair loss and induce hair follicle redevelopment.

## 7. Conclusions

The scRNA-seq technology can identify cell genetic information from the single cell level, which provides a powerful tool for identifying the transcriptome characteristics of various cell types in heterogeneous populations. With the continuous progress of single-cell sequencing research, we are unveiling the veil of cell fate selection and life occurrence. Analysis of cell genomes or transcriptomes at the single cell level has enabled biological research to reach unprecedented levels of resolution and scale, which is transformative in extremely complex hair follicle systems. Indeed, scRNA-seq can provide new perspectives and methods for life science research. The continuous development of scRNA-seq and its combination with multi-domain technologies and algorithms will bring a new revolution to next-generation genome sequencing. Together this will become an important tool for studying the type and state of hair follicle cells and help to develop reasonable methods for the treatment of future hair loss diseases, as well as to analyze the mechanism of hair follicle morphogenesis.

## Figures and Tables

**Table 1:** Markers for identification of major cell types in hair follicles.

Cell Type	Markers	Sample Source
Dermal papilla	SOX2 [53,54], SOX18 [53,55], LEF1 [53], CORIN [53,56], FGFR1 [53], WNT5A [53], WIF1 [54], LEPR [54,57,58], HHIP [59], VCAN [59], MDK [59], DRAXIN [59], NOTUM [56]	Mice [53,54,55,56,57,58,59]
Hair shaft	MSX1 [60], LHX2 [60], HOXC13 [60], FOXQ1 [60], GRHL1 [60], ACPP [60]	Mice [60,61]
Melanocyte	PLP1 [61], FABP7 [61], DCT [54], MITF [54], HSDT7B12 [59], NUDT17 [59], PMVK [59], MLANA [62]	Human [62]
Outer root sheath	SOX9 [60], LHX2 [60], FOXE1 [60], TAGLN [63], SLC1A3 [63], FGF5 [63], PTHLH [56,63], WFDC18 [56,63]	Mice [63]
Inner root sheaths	NRP2 [60], KRT71 [56,64], KRT28 [56,64], KRT27 [56,64], KRT25 [64,65]	Human [64,65]
Hair follicle Stem cells	SOX9 [54,63,66,67], LHX2 [54,66,67], NFATC1 [54,66], LGR6 [54], CD34 [63,68], LCR5 [63], KRT14 [63], TCF4 [67]	Mice [66,68], Human [67]
Hair matrix	SHH [54], MSX2 [54,69], LHX2 [70], FOXN1 [69]	Mice [69,70]
Endothelial	TIE2 [54,63], CD31 [54,55,63], CDH5 [63], VEGFR1 [63]	Mice [54,55,63]
Dermal sheath	ACTA2 [58], TAGLN [58], MYLK [58], RAMP1 [58], COL11A1 [62], ACAN [62], HES1 [62], MYL4 [62], CTNNB1 [62]	Mice [58], Human [62]
Fibroblasts	CRABP1 [55], FABP5 [55], RUNX1 [55], CD26 [55], SCA1 [55], PDGFRA [63], VIMENTIN [63], COL1A2 [62,71], DCN [56,62,71], LUM [62,71], PDGFRA [62,71], VIM [62,71], DPP4 [72], GPX3 [56], SPARC [56], PLAC8 [56]	Mice [55,56,63], Human [62,71,72]
Interfollicular epidermis	LMO1 [66], WNT4 [66], THBS1 [73], KRT14 [73,74], KRT5 [73], MT1 [73], MT2 [73,74], KRT10 [73,74], SBSN [73], MT4 [73], IVL [73], FLG2 [73,74], LOR [73,74], PTGS [74], KRT1 [73], KRT17 [73]	Mice [66,73,74]
Bulge	DAPL1 [63], THEM5 [63], BDNF [63], ANK [63], POSTN [74], CD34 [74], KRT15 [64,66,74]	Mice [63,66,74], Human [64]
Infundibulum	MKI67 [73], TUBB5 [73], TOP2A [73], UBE2C [73], FST [74], AQP3 [74], SOSTDC1 [74]	Mice [73,74]
Upper hair follicle	KRT79 [74], KRT17 [74], LOR [74], FLG2 [74], KRT10 [74], PTGS1 [74], PTN [74], LRIG1 [74], DEFB6 [74], CST6 [74]	Mice [74]
Keratinocytes	KRT1 [71,75,76], KRT10 [71,75,77], OVOL1 [76], EVPL [76], KRT14 [58,77,78], S100A2 [79], KLK7 [80]	Human [71,75,79,80], Mice [58,76,77], Goat [78]

## Data Availability

Not applicable.
